# A new public health context to understand male sex work

**DOI:** 10.1186/s12889-015-1498-7

**Published:** 2015-03-24

**Authors:** Victor Minichiello, John Scott, Denton Callander

**Affiliations:** Australian Research Centre in Sex, Health and Culture, La Trobe University, Melbourne, Australia; University of New England, Armidale, Australia; School of Justice, Faculty of Law, Queensland University of Technology, Brisbane, Australia; Kirby Institute of Infection and Immunity in Society, University of New South Wales, Sydney, Australia

**Keywords:** Male sex worker, Male sex industry, Sexualities, e-technologies, HIV/AIDS

## Abstract

**Background:**

Researching male sex work offers insight into the sexual lives of men and women while developing a more realistic appreciation for the changing issues associated with male sex work. This type of research is important because it not only reflects a growing and diversifying consumer demand for male sex work, but also because it enables the construction of knowledge that is up-to-date with changing ideas around sex and sexualities.

**Discussion:**

This paper discusses a range of issues emerging in the male sex industry. Notably, globalisation and technology have contributed to the normalisation of male sex work and reshaped the landscape in which the male sex industry operates. As part of this discussion, we review STI and HIV rates among male sex workers at a global level, which are widely disparate and geographically contextual, with rates of HIV among male sex workers ranging from 0% in some areas to 50% in others. The Internet has reshaped the way that male sex workers and clients connect and has been identified as a useful space for safer sex messages and research that seeks out hidden or commonly excluded populations.

**Future directions:**

We argue for a public health context that recognises the emerging and changing nature of male sex work, which means programs and policies that are appropriate for this population group. Online communities relating to male sex work are important avenues for safer sexual messages and unique opportunities to reach often excluded sub-populations of both clients and male sex workers. The changing structure and organisation of male sex work alongside rapidly changing cultural, academic and medical discourses provide new insight but also new challenges to how we conceive the sexualities of men and male sex workers. Public health initiatives must reflect upon and incorporate this knowledge.

## Changing sexuality and the normalisation of male sex work

Is the male sex industry a reality or fiction? This question might seem a strange way to begin, but it helps provide important context for this article. When society talks about the sex industry – mainly through discourses of popular culture – it is most commonly through the archetypes of women as sellers and men as buyers that these issues are cast. Yet, Internet technologies have made one thing very clear: there is a sizeable section of the sex industry where men are the sellers. Recently, our edited collection, *Male Sex Work and Society*, provided an up-to-date account of some of the key topics associated with male sex work (MSW) [1]. This collection, alongside an increasing number of diverse international studies, highlights several important points about the male sex industry. First, the phenomenon of MSW has ‘global’ visibility. MSW exists in all societies and researchers have recently recognised the diverse structural and organisational aspects of MSW as it is practiced in various locations [1]. Second, by all accounts there remains a strong demand for male sex workers (MSWs) in many parts of the world [1]. Third, the growth and evolving shape of the male sex industry has challenged traditional or hegemonic gender constructs. For example, men are no longer the exclusive consumers of sex and the male body can be represented as a desirable commodity for others. And finally, although contemporary constructions of sex work have moved beyond sex workers as mere vectors for disease transmission, MSWs still represent a population at-risk for HIV and other sexually transmissible infections (STIs). The purpose of this paper is not to offer a comprehensive review of the research on MSWs (we have previously reviewed the literature in this area [2]) but instead to highlight some evolving and key issues of interest to the public health field and society broadly. It is our contention that effective public health research and policy agendas must make use of the shifting face of MSW and draw deeper into the arenas of lifestyle and sexuality.

It is first necessary to recognise the complicated language that surrounds the sex industry and sex work. ‘Sex work’ is used over ‘prostitution’ because it helps challenge the long history stigmatisation associated with being a prostitute and evokes important notions of industry and employment [3]. The expression ‘male sex worker’ is used in this paper to refer to sex workers whose sex as determined by biological markers was deemed male at the time of birth. This nuance deliberately excludes transgender sex workers in recognition of the different issues faced by that population. Most of the research cited in this article adopted this same definition of MSW and instances where this was not so have been made explicit. It is also necessary to recognise that sex work is often organised – particularly in research – according to location and we have therefore considered the places or contexts in which MSWs operate, such as on the street, in brothels, or online.

It is important to recognise that sex work among men is not a new phenomenon. Friedman, for example, reviewed the cultural conditions of MSW as it existed in ancient Greece and Rome, pre-modern and Renaissance Europe, and Japan in the days of the samurais [4]. It is equally important to recognise that MSWs are not a homogenous population. Research has found significant differences between street, escort and brothel sex workers with respect to types of services offered, education, age, sexual identity, race [5,6]. Globally, research has also found vast differences in income and rates of HIV among MSWs [5,7], further highlighting the diverse experiences and situations relevant to this population. Research has also shown that physical positioning, based on masculine norms – top (insertive/dominant) or bottom (receptive/submissive) – plays some part in determining what MSWs charge their clients [5]. It has also been reported that masculinity along with sexual identity can also influence safer sex behaviours among MSWs [8,9].

In the past century, sex work has represented the archetypal form of gendered deviance in modernity, being variously problematised as a criminal activity and a source of contagion [10]. As we have observed elsewhere, MSW itself has not always been considered socially problematic [2] with MSW historically characterised as a ‘deviant’ social behaviour not because of the sale of sex but because of the association with male homosexuality. Social concern around MSW also focused on the challenges it presents to gender norms and hegemonic forms of masculinity. For example, MSW, by definition, presents the male body as a commodity and in doing so may confront expressions of masculinity that rely on agency or aggression. MSW also presents challenges to class and generational boundaries, as seen through the ideas of ‘rough trade’ (slang for working-class men who had sex with other, usually wealthier, men for money) and ‘kept boys’/’sugar daddies’ (slang, an older man financially supports a younger man, which may involve sexual or romantic components) [10]. Female sex work has largely been viewed within feminist theories as a gendered institution, which oppresses women while explicitly exploiting them for male consumption [11]. Female sex work, however, also raises questions about agency, sexual liberation and empowerment, which is why some earlier writing referred to it as a ‘dilemma’ for feminist theory [12].

Although MSW remains criminalised in many parts of the world, questions of its normalisation are warranted. In post-structuralist and post-modernist conceptualisations of risk, the construction of ‘deviance’ is a consequence of social groups increasingly encountering one another as risks [13]. As mentioned, MSW has historically been viewed as deviant because of its association with homosexuality so the decriminalisation of homosexuality in many countries may have resulted in less policing, both socially and legally, of MSW [2]. Kaye has argued that new social meanings for MSW in the 1970s were derived through the, “progressive integration of male prostitution into the gay cultural orbit” [14] and there have been legislative reforms that allow gay men and women to present more visibly as social groups and communities. This change helped facilitate the appearance of more gay-identified men in samples of MSWs in the USA, with some men reporting a positive and professional approach to their work [15]. For the first time, researchers began to challenge the constructions of male prostitution as a criminal (MSWs as victim) or health (MSWs as agent) problem. Recent research suggests that MSWs who identify as gay or bisexual tend to identify sexual pleasure as an important aspect of their work along with other positive work related experiences, such as earning ability, flexibility of work, skill development and client satisfaction [16].

There have also been important social shifts regarding masculinity. In recent years, masculinity and male bodies have in been openly represented in eroticized cultural terms in art, fashion and film, which have worked to change popular thinking about male sexuality and masculinity [17]. Giddens’, for example, has spoken about ‘plastic sexuality’ [18] to emphasise the way in which sexuality has been decentred in recent decades, having been freed from its links with reproduction. Bauman’s arguments around ‘liquid love’ highlight the weakening of traditional bonds such as marriage and familial relations and how notions of love have become commoditized in recent decades [19]. Specific to MSW, the fluidity that this introduces to sexual identities and relationships may contribute to willingness for diverse types of men to engage in sex work, which can be seen, for example, through the growing phenomena of straight-identifying men engaging in same-sex work or pornography, known as ‘gay for pay’ [20].

The growing fluidity of sexual experiences is further highlighted by the declining currency of sexual dichotomies like heterosexual/homosexual. Indeed, the pluralisation of ‘sexualities’ is further evidence of this point. The idea of ‘gay for pay’ is but one manifestation of this social trend and it carries with it significant public health implications for the male sex industry: interventions that target a homogenous group of gay men as workers or clients may no longer be wholly valid. For example, our work with MSWs in Argentina found that men of diverse sexualities make complex decisions about working as a sex worker and providing sexual services to both men and women [21]. Likewise, our review of the online profiles of male clients of MSWs revealed that these men are not easy to stereotype: they are young, old, blue-collar workers, professionals, gay, bisexual, straight, married, in relationships, bachelors, fit, overweight, homely, and handsome [22]. This diversity highlights the many different kinds of experiences that are maintained by both clients and workers, not to mention varying degrees of victimization, exploitation, agency and choice.

While shifting social mores have had a role in the normalization of MSW – especially in terms of social responses to same-sex attraction – it also clear that material conditions have impacted on the way in which this industry is structured and organised [2]. Here we highlight and discuss the impact of globalisation as witnessed via the increase mobility of MSWs, particularly from countries like the USA, Canada and Europe, to travel within and between countries, partly as a result of the emerging Internet technologies has changed the face of the male sex industry.

## Review

### A global reality

Globalisation has provided the male sex industry with unprecedented visibility, especially in terms of cultural diversity [23,24]. Increasingly, research on MSW has highlighted global nuances around expressions of masculinity, the social control of gendered deviance, and sexual practices. Notably, more and more research and journalism has rightly recognised that MSW is not an exclusively Western phenomenon. Peter Aggleton’s collection of essays, *Men who Sell Sex,* was the first serious attempt to present a global account of MSW and featured research from South America, South Asia and Southeast Asia and [25]. Others have also sought to highlight the global nature of MSW. Alcano, for example, provided one of the first contemporary accounts of MSW markets in Indonesia through a vivid account of how masculinity is uniquely constructed, enacted and reproduced in a South East Asian setting [26]. Similarly, Mitchell examined masculinities with regard to Brazilian rent-boys, exploring how ‘Latin homosexuality’ mediated MSW encounters, especially with regard to the adoption of passive or active roles in a sexual encounter [27].

Globalisation and MSW has also been explored with respect to ‘sex tourism’, which is typically thought of as the use of the tourism sector to facilitate commercial sexual relationships with local residents. In the past decade there has been interest in this topic from a variety of positions related to the male sex industry. One study of MSWs and their male clients in the Caribbean explored the concept of authenticity regarding these relationships [28]. Sex tourism has also been cast as a public health issue, particularly in the Caribbean where rates of HIV are among the highest in the world [29,30].

Sex tourism in the Caribbean is also a somewhat unique context for the study of MSW because it is one of the areas to spark research on female clients. One study from the Dominican explored the transactional relationships that predominantly White Western women formed with local men, which often involved the indirect provision of money and goods [31]. Although some earlier work suggested that women were actually looking for romance through these encounters and not sex (leading to the idea of ‘romance tourism’) [32], others have noted that women in these situations have diverse desires and expectations [31]. These encounters generate a complex intersection of class, power, race and gender, which has also been cast as ‘post-colonial’ as local men and female tourists both seek out opportunities for self-realisation and liberation through the connections they form [33]. In some cases, the traditional order often invoked through notions of class, race and gender appear to be suspended by these ‘transgressive’ relationships [34]. These explorations are quite unique and offer new insight into the male sex industry while raising questions about how we perceive the relationship between client and worker and, importantly, how researchers characterise clients that are female.

The globalisation of MSW demands nationally and culturally contextualised public health campaigns. For example, many men who engage in transactional sex in the Caribbean – particularly with female clients – do not self-identify as sex workers [31]. Any attempts to reach this population must consider this nuance. Regarding masculinity, while MSW in some parts of the world may challenge identity and manhood, in other cultures of MSW this stigma is managed through an emphasis on hegemonic models of masculinity [26]. These are key differences that highlight the force of globalisation with regards to both the male sex industry and the associated public health issues.

### Sexual health and male sex workers

Although HIV and STIs are not issues unique to MSWs, they remain an important aspect of how we understand this population. It is necessary to recognise, however, that there is no static or uniform rate of HIV/STI infection among MSWs. We must also consider the cultural context of sex work, as markers of prevalence are globally quite varied. Methodologies, definitions of sex work, and broader norms around sexuality often confuse direct comparisons across the large body of work in this area. Even within geographic localities, rates can vary widely. Sampling of sex workers is also a key issue, particularly because, as we have discussed, MSWs do not form a neatly homogenous population. It is, therefore, necessary to exercise caution when interpreting rates of infection and reported risk behaviours. Nevertheless, sexual health epidemiology can provide an important perspective on MSW.

In this section, we report on a sample of recent work detailing HIV and, where available, STI epidemiology among MSWs, which has been organised (mainly) along continental lines. Peer-reviewed journals and reports were sourced using keyword searches via academic databases, such as Google Scholar, Scopus and PubMed. Search terms included: male sex work*; MSW; sexually transmissible infect*; STI; HIV; chlamydia; gonorrhoea; syphilis; sexual risk; condoms; sexual health. Wherever possible the cited research has been limited to studies conducted in the last decade (2004 onward) with a few exceptions where more recent literature was not available. Across a diverse cohort of studies, rates of HIV among MSWs have been reported as between 0 – 50% while rates of STIs range from <1 – 60%. Table 1 provides a full overview of studies that provide an estimate of HIV/STI prevalence and incidence among MSW. It also highlights some studies that provide estimates around consistent use of condoms with MSWs and their clients.

**Table 1:** **A global perspective on HIV and STI prevalence and consistent condom use among male sex workers**

			**MSW prevalence**		**Consistent condom use** **	**Recruitment**
	**[#] Authors**	**Year**	**HIV**	**STIs** *		
**Africa**						
Côte d’Ivoire	[35] Vuylsteke et al.	2012	50%	NG^†^ (13%)	69%	MSW clinic
				CT^‡^ (3%)		
Kenya	[97] Sanders et al.	2007	43%	--	--	MSW venues
	[98] Mwangome et al.	2009	8.6py^§^	--	--	
	[38] Geibel et al.	2008	--	--	36%	MSM venues
	[37] Muraguri et al.	2014	26%	NG rectal (6%)	--	HIV organisation
				NG urethral (5%)		
				CT rectal (3%)		
				CT urethral (3%)		
				Syphilis (2%)		
**Asia**						
India	[99] Shinde et al.	2009	33%	Any (60%)	33%	HIV/STI clinic
	[41] Narayanan et al.	2013	43%	NG rectal (10%)	82 – 88%	HIV/STI clinic
				CT rectal (5%)		
				Syphilis (8%)		
	[100] Solomon et al.	2010	9%	Syphilis (8%)	55%	RDS^¶^
China	[42] Yu et al.	2013	6%	--	74%	MSW venues
	[101] He, et al.	2007	3%	Any (29%)	50%	RDS
	[102] Cai, et al.	2010	5%	Syphilis (14%)	37%	MSW venues
	[43] Chow et al.	2012	2 – 12%	Syphilis (5-20%)	15 – 95%	Meta-analysis/review
	[9] Wong et al.	2008	2%	Any (9%)	64%	RDS/SB^\\^
	[44] Huan et al.	2013	11%	Syphilis (28%)	--	RDS
Indonesia	[45] Pisani et al.	2004	4%	Syphilis (2%)	35%	MSW venues
Thailand	[46] Toledo et al.	2010	19%	Any (66%)	82%	MSW venues
Pakistan	[47] Mumtaz et al.	2011	0 – 6%	--	--	Review
	[48] Hawkes et al.	2009	0%	NG rectal (5 – 20%)	10%	RDS
				CT rectal (5 – 10%)		
Uzbekistan	[49] Todd et al.	2007	6%	--	2%	Community organisation
Israel	[50] Mor & Dan	2012	5%	NG throat (2%)	40%	MSM venue/SB
				NG urethral (<1%)		
				CT urethral (2%		
				Syphilis (3%)		
Russia	[52] Baral et al.	2010	18%	Syphilis (12%)	71%	MSW venue
			MSW prevalence		Consistent condom use**	Recruitment
	[#] Authors	Year	HIV	STIs*		
**Central and South America**						
Peru	[53] Bayer et al.	2014	18%	Syphilis (8%)	41%	MSW venue
Brazil	[57] Cortez et al.	2011	17%	--	68%	SB
**Europe**						
Belgium	[59] Leuridan et al.	2005	11%	NG urethral (3%)	79%	Community organisation
				CT urethral (10%)		
				Syphilis (13%)		
UK	[61] Sethi et al.	2006	9%	NG (9%)	96%	MSW clinic
				CT (7%)		
				Syphilis (21%)		
	[62] Mc Grath-Lone et al.	2014	4%	NG (17%)	--	Men’s health clinics
				CT (25%)		
				Syphilis (3%)		
Spain	[66] Belza	2005	12%	--	--	HIV/STI clinic
Germany	[67] UNAIDS	2012	5 – 10%	--	--	Surveillance data
**Australia and North America**						
Australia	[68] Estcourt et al.	2000	7%	NG (5%)	86%	MSW venues
				CT (2%)		
				Syphilis (3%)		
	[69] Minichiello et al.	2002	7%	--	95%	MSM venues
	[103] Prestage et al.	2007	--	--	80%	MSM venues
	[104] Wilkinson et al.	2012	--	CT (14.3py)	--	Surveillance data
USA	[71] Timpson et al.	2007	26%	--	--	MSW venue/SB
	[72] Smith & Seal	2008	--	--	97%	MSW venue
Canada	[75] Weber et al.	2001	5%	--	--	MSM venue

*Where anatomical site is not specified it was not clear where samples were collected.

**Wherever possible, condom use is reported between MSWs and their clients and limited to anal sex.

^†^NG: Neisseria gonorrhoea.

^‡^CT: Chlamydia trachomatis.

^§^py: Person years (calculated per 100).

^¶^RDS: Respondent driven sampling.

^\\^Snowball sampling.

Estimated rates of HIV are highest among MSWs in African countries, which is unsurprising given the disproportionate global burden placed upon this continent. Within Africa, however, rates appear to be significantly higher among MSWs compared with the general population of gay, bisexual and other men who have sex with men (GBM). In Cote d’Ivoire and Kenya it has been reported that MSW have significantly higher rates of HIV than GBM [35,36], although one Kenyan study found no difference in STI prevalence [37]. Importantly, one study found that 35% of MSW did not know HIV could be transmitted via anal sex [38] and it has been reported that clients in parts of Africa sometimes pay less for sex with a condom or will behave violently towards MSWs who refuse condomless sex [39].

Across the Asian continent, the largest body of MSW research comes from India and China. In India is has been reported that risk of infection as well as rates of HIV are twice as high among MSW as they are among GBM generally [40,41]. By contrast, HIV among Chinese ‘money boys’ (a slang term for MSWs in China) has, in some research, been reported as lower than that of the general GBM population (6% vs 10%, respectively) [42]. This difference could be partly explained by the findings of a meta-analysis that money boys are more likely to use condoms during anal sex than GBM generally [43]. Identity may also factor into these dynamics, as past research has reported that gay-identified money boys were tested for STIs and HIV more frequently than their non-gay identified peers but also took more sexual risks [9]. That study also reported that 60% of the money boys sampled did not know their HIV status. Other work has found that sex work predicted a greater likelihood of syphilis infection among GBM in China [44].

Elsewhere in Asia, rates of HIV and STIs among MSW range widely. In Indonesia, 4% of MSWs were found to have HIV and 2% had syphilis, which compared with 3% and 1% among GBM generally [45]. Rates were markedly higher in a Thai study, which found an overall MSW HIV prevalence of 19%, with significant differences between men who worked in entertainment venues (16%) and those who worked on the street (23%) [46]. In Pakistan, there is no available epidemiological data on HIV among GBM, although rates among MSWs have been estimated between 0 – 6% with 1 – 14% ever tested for HIV [47,48]. Nearby in Uzbekistan, HIV prevalence among MSWs has been reported at around 6% with only 2% reporting consistent condom use with clients [49]. In Israel, HIV and STI prevalence appears to be much higher among MSWs than GBM generally [50,51]. Finally, HIV prevalence in Russia has been reported as 18% among MSWs with 25% of that sample infected with one or more other STI [52].

Turning to Central and South America, MSW in Peru appear to have slightly lower rates of HIV than GBM generally with observed differences in prevalence between men from downtown urban areas and those from nearby neighbourhoods (23% vs 4%) [53,54]. MSW in downtown areas also had higher rates of condomless sex with clients [55]. In the Dominican, interviews revealed largely inconsistent condom use among MSW and their clients [56]. And a Brazilian study reported a 17% HIV prevalence rate among MSWs in that country [57], which compares with 12% reported among GBM broadly [58].

European research further highlights the wide differences in HIV and STI rates that span a geographically close space. In one study from Belgium, 11% of MSW were diagnosed with HIV [59], which is comparable to another sample of GBM generally [60]. In the UK, somewhat lower rates of HIV among MSW have been reported [61,62], though they were also similar to those among GBM generally [63-65]. Slightly higher prevalence of HIV has been reported among MSW in Spain (12%) [66] while rates appear to be lower in Germany (5 – 10%) [67].

Finally, North American and Australian rates of HIV among MSWs are relatively low. Australian work has suggested an HIV prevalence rate of around 7% of MSW in urban areas [68,69], which compares with 13% among a more recent survey sample of urban GBM [70]. A study from Houston in the USA reported a 26% prevalence of HIV among MSWs there [71]. It has been reported, however, that MSWs in the USA generally do not engage in sexual risk-taking with their clients [72,73]. Recent Canadian research with young MSW, reported high demand for condomless sex from sex work clients [74]. Another study of young MSWs in Canada found significantly higher rates of HIV when compared with other GBM (5% vs 1%) [75].

Around the world, rates of HIV and other STIs among MSW vary widely and while some countries have no or very little incidence, others face a major epidemic. The same can also be said of MSWs sexual risk behaviours, with men in some parts of the world negotiating consistent condom use while others may even be unaware of the risks condomless sex can pose. There are also many mediating factors for risk and behaviour, some of which have been hinted at here. Violence, poverty, substance use, identity, and knowledge all play a major part in the sexual health of MSWs. Returning to the nature of globalisation, local laws and cultural norms also play into the health of MSWs. As Baral and colleagues point out, fear of persecution in places where sex work or same-sex relations are illegal or stigmatised reduces service accessibility for this population, which exacerbates their risk for HIV and other STIs (not to mention onward transmission) [76]. Assessing the epidemiology presented here requires full consideration for these overlapping issues.

### The impact of Internet technologies

As noted, the expansion and availability of online technologies have assisted in the normalisation of MSW. Not only does the Internet provide unprecedented opportunity for public to access information on MSW but it can also make access to the male sex industry easier (and safer) for both clients and workers. Through the Internet, individuals can access the virtual world of MSW from the comfort of their living rooms and do so with a high degree of anonymity. These features not only foster discretion but they also provide a safer environment that is less affected by the risks of robbery, blackmail or violence so often associated with street sex work. Client screening websites (e.g., http://www.daddysreviews.com) provide MSWs with an opportunity to review clients and alert peers to personal risks. Conversely, other websites allow clients to share their reports on sex workers. For example, one website used to review female sex workers, “The Erotic Review” (http://www.theeroticreview.com), has between 500,000 to 1 million unique visitors monthly [77]. Forums such as these are examples of how a previously solitary behaviour is made public through virtual spaces [78], which highlights the simultaneously public and private nature of online engagement.

The Internet has also meant that MSW and associated information can reach an audience with greater socio-demographic diversity than through other sex work venues [78-80]. Beyond the ability of the Internet to flatten social and geographical spaces, the aforementioned privacy that it affords may help make MSW more accessible to certain populations, such as men who may have sex with men but do not self-identify as gay or bisexual [81,82]. Indeed, some research from the USA highlights that many MSWs serve a sizeable market of clients who identify as straight, with the geographic distribution of MSWs following metropolitan populations and not the location patterns of gay communities [5].

With respect to sexual health and MSW online, the virtual communities described here may provide new avenues for disseminating safer sexual messages to MSW and their clients [83,84]. Of course, it is also necessary to consider if or how these communities may be at risk for HIV or other STIs. While some research has found that MSWs operating online do not specifically seek-out condomless sex [85], a review of online profiles posted by MSWs found that nearly half were ambiguous about their safer sex intentions or indicated a willingness to have condomless sex [8]. The question, of course, is if MSWs and clients who organise online exhibit different sexual risk behaviours than other sex worker groups. One study that compared MSWs who meet clients on the street to those who meet clients online found that online MSWs tended to have more clients on average but also found that both groups reported inconsistent condom use with their clients [86]. While other studies among men who have sex with men report that the Internet may create spaces for men to organise condomless anal sex [87,88], it is less clear if similar negotiations are taking place among MSWs and their clients.

For MSW, the Internet has played a significant part in coalescing previously disparate communities. As a result, public health research and strategy should consider how to capitalise on these connections to distribute safer sex messages and access hard-to-reach populations, particularly women (who are so often missed in the literature in spite of evidence of their growing part in the male sex industry) and men who do not identify as gay or bisexual. The Internet undoutabley has a part to play in this research. A recent survey of 258 online escorts in Australia, for example, identified a number of escort agencies that catered exclusively for a female clientele and 35 escorts provided specific services for women; of the escorts who specified sexual preference in their online profile, 33 described themselves as bisexual, 22 as gay and 38 as straight [89]. The Internet also provides examples of articles and testimonials shared by female clients, providing some insight into their experiences and perceptions [22]. More rigorous work is needed, however, to explore not only the women themselves who hire MSWs but also how masculinity, gender, power and safer sex are negotiated through these encounters.

Finally, the Internet has made pornography much more accessible. The connection between MSWs and pornography is complex and under-researched. Some researchers are opposed to both pornography and the sex industry on the grounds that the industry is inherently harmful to women, as it is exploitative and that its consumption eroticizes domination and humiliation [90]. It is not clear, however, whether the same argument of exploitation holds true with the male sex industry. Some could speculate that the experiences of men and women in these industries may be different and that male performers (as porn stars or sex workers) enjoy greater agency, or that the sexual commercialisation of men does not reproduce inequality in the same way that it does for women. Further connecting MSW to pornography is the reality that some MSWs also work as porn actors. This relationship has given rise to new types of ‘pornographic services’ offered by MSWs, such as live online sex shows. Regarding safer sex, in the face of the growing popularity of condomless sex in gay pornography [91], it is important to question how these representations of sex may influence the desires and demands of clients as well as the services on offer from MSWs.

## Conclusions

New information technologies have assisted in developing a ‘global’ perspective on MSW sensitive to cultural variations in the expression of masculine norms. Information technologies have also changed the way in the sex industry is organised and structured, and assisted in the development of a collective response among the sex industry to issues that have marginalised sex workers as dangerous criminals. The political organisation of sex workers since the 1970s has been aimed at promoting the human rights of sex industry workers alongside issues of occupational health and safety. During the same period civil libertarian groups and some feminist organisations allied themselves with sex workers to lobby for legal reform. More recently, various sex worker rights organizations have attempted to coordinate such activities, setting up the International Committee on Prostitute’s Rights. While much of this work was centred on female sex work by specifically targeting associated gender biases and stigma, MSWs have also been politically active with their work best understood through a prism of the evolving gay rights movements. Recent MSW-specific advocacy and support groups include Hook (Online), the Global Network of Sex Worker Projects, Sex Worker Education and Advocacy Task (South Africa), The Asia Pacific Network of Sex Workers, Scarlet Alliance (Australia), Sex Professionals of Canada, The Network of Sex Workers (Latin America), and the European Network on Male Prostitution. The Internet has strengthened efforts to coordinate the political activity of sex workers nationally and internationally [92].

While in the past MSW was constructed as pathological or deviant, research is now documenting much more clearly how MSW unfolds and evolves as part of some people’s everyday experience. Much current research has re-framed MSW according to an occupational perspective that argues MSW can be a rationally chosen and satisfying activity for both client and MSW. As a result, we can better conceptualise and capture these transactional moments of sexual intimacy between MSWs and their clients with greater accuracy, realism and sympathy [93]. From a public health perspective, however, the criminalisation of same-sex relationships and sex work hinders the translation of these theoretical conceptualisations into improved health outcomes. Criminalisation of sex work not only creates a space rife for violence and sexual health risk [94] but it also challenges the basic implementation of health and safety initiatives by interfering with their successful operation. It has been reported, for example, that carrying condoms has been used as an excuse to harass female sex workers in the USA [95]. Avoiding social sanctions such as these means that sex workers remain hidden from public view, which challenges important work around disease surveillance and outreach initiatives [96]. Confronting state-sanctioned stigma and discrimination is a key component of more a more effective public health approach for both MSWs and their clients.

Safer sex must be negotiated alongside fantasies, intentions, motivations, power, and desire. In some cases the desire for intimacy and fulfilment may exceed the desire for protection. In others, MSWs and clients may seek expressions of masculinity and dominance by penetrating without condoms. In some situations, MSWs may be unable to negotiate condom use due to threats of violence or lost revenue. Further, as pre-exposure prophylaxis (“PrEP”) is increasingly touted as a major component in the international effort to combat HIV, particularly among high-risk populations, the stage is set for new questions about how this medication will be used by or provided to MSWs in diverse contexts. Research must therefore move beyond identifying MSWs and clients and seek instead to better understand the human dimensions that underpin such relationships. This approach will help inform the development of more effective public health policies and interventions.

This article’s discussions and arguments should be considered in the context of their limitations and strengths. First, epidemiological comparisons of HIV or other STIs among MSWs are not always appropriate given vast global differences in reporting requirements, not to mention the potential stigma or legal redress associated with identifying as a ‘male sex worker’. Second, this paper is an overview of key issues defining MSW at the moment and not a comprehensive review of the available literature. Finally, comparisons between male and female sex workers and their male and female clients are always challenged by the very different ways that these groups are thought about in and constructed by society and academia. Although we have drawn comparative arguments throughout, far more work is required to systematically unpack the similarities and differences.

This article comprehensively reviews sex work among men by accounting for how the globalisation of MSW through changes in society, legality and technology. By providing an overview of HIV and STI burden among members of this population, this paper contributes important epidemiological information relevant to advocacy, outreach and health promotion. Consideration for the contemporary paradigms in which MSW now operates, this paper is well-situated to continue the discussion around MSW and help foster more nuanced and targeted public health initiatives. With women increasingly visible as clients and with the demographic diversity of men who buy and sell sex, it is clear that there is need for a major rethink of how MSW is regulated and professionalised to ensure appropriate public health services and outcomes for both MSWs and their clients.
